# Death anxiety predicts fear of Cancer recurrence and progression in ovarian Cancer patients over and above other cognitive factors

**DOI:** 10.1007/s10865-023-00422-w

**Published:** 2023-06-12

**Authors:** D Coutts-Bain, Louise Sharpe, H Russell

**Affiliations:** 1 School of Psychology, The University of Sydney, Sydney, NSW 2050 Australia; 2 Ovarian Cancer Australia, Melbourne, VIC Australia

**Keywords:** Fear of cancer recurrence, Fear of progression, Death anxiety, Cognition, Psycho-oncology, cancer

## Abstract

Death anxiety is understudied in people with cancer, especially in relation to fear of cancer recurrence (FCR) and fear of progression (FOP). The present study aimed to identify if death anxiety can predict FCR and FOP over and above other known theoretical predictors. One hundred and seventy-six participants with ovarian cancer were recruited for an online survey. We included theoretical variables, such as metacognitions, intrusive thoughts about cancer, perceived risk of recurrence or progression, and threat appraisal, in regression analyses to predict FCR or FOP. We investigated whether death anxiety added to the variance over and above these variables. Correlational analyses demonstrated that death anxiety is more strongly associated with FOP than FCR. The hierarchical regression including the theoretical variables described above predicted 62–66% of variance in FCR and FOP. In both models, death anxiety predicted a small but statistically significant unique variance in FCR and FOP. These findings draw attention to the importance of death anxiety in understanding FCR and FOP in people with a diagnosis of ovarian cancer. They also suggest that elements of exposure and existentialist therapies may be relevant in treating FCR and FOP.

Ovarian cancer is often diagnosed at advanced stages of disease (Torre et al., 2018). Consequently, whilst treatment can be effective in the short term, approximately 70% of people with ovarian cancer will experience recurrence, and only 46% of people survive five years beyond initial diagnosis ((AIHW), 2020; Jelovac & Armstrong 2011). Hence, it is unsurprising that fear of cancer recurrence (FCR), fear of progression (FOP), and death anxiety are significant concerns for people living with ovarian cancer (Tan et al., 2021).

FCR is most commonly defined as a “fear, worry, or concern that cancer will recur or progress” (Lebel et al., 2016). However, recent research has demonstrated that whilst FCR and FOP are related, they are in fact distinct constructs (Coutts-Bain et al., 2022). Specifically, Coutts-Bain and colleagues demonstrated that two of the most validated measures of FCR and FOP load onto separate, albeit related, factors. Moreover, FCR and FOP are not entirely predicted by the same theoretical constructs. Drawing from the avoidance model of worry (Sibrava & Borkovec, 2006), it was postulated that FCR may in fact represent cognitive avoidance of FOP. In this account, worry about recurrence and symptoms indicative of recurrence avoids mental imagery and anxiety about what occurs after recurrence, namely progression of the disease leading to death. Although the prevailing definition of FCR conflates FOP and FCR, in this paper FCR is defined as the fear that cancer will recur, and FOP as the fear that cancer will progress.

Whilst these fears are an expected and normal part of adjusting to life with cancer, which could be adaptive by motivating health behaviours and vigilance towards signs of recurrence and progression, these fears can become chronic and distressing in and of themselves (Herschbach & Dinkel, 2014; Lee-Jones et al., 1997; Simard et al., 2013). Indeed, greater FCR and FOP have been associated with higher rates of psychological outcomes and poorer quality of life (Dinkel et al., 2014; Simard et al., 2013). There is also evidence that higher levels of FCR are actually associated with maladaptive health behaviours. High FCR is associated with increased tobacco use in head and neck cancer survivors (Van Liew et al., 2014), and reduced use of mammograms and ultrasounds in female breast cancer survivors (Thewes et al., 2012). Hence, despite a growing research base, research on models of FCR and FOP intervention and mechanisms of action are still recognised as critical areas in need of further research (Shaw et al., 2021). In this spirit, death anxiety may be a novel factor relevant to the treatment of FCR and FOP (Sharpe et al., 2018).

Death anxiety is a specific existential concern whereby one has a desire to continue to live, but is nevertheless aware of their own mortality (Yalom, 1980). And, as previously alluded to, existential issues, such as death anxiety, are inextricably linked to cancer-related anxieties, including FCR and FOP (Curran et al., 2017). Whilst other existential concerns exist, such as meaningless, which is an inner-conflict arising from a desire to believe that life has a central meaning despite existing in a world which appears random or inconsistent with established meanings (Koole et al., 2006), arguably death anxiety is most central to FCR and FOP. If one holds an an innate desire to maintain one’s life, then cancer, particularly ovarian cancer, represents a substantial threat to life and reminder of mortality. This is intuitive, and has been identified by qualitative research (Moskalewicz et al., 2022; Thewes et al., 2016). Despite this, there is scant quantitative research on death anxiety as it relates to FCR or FOP, beyond a few studies showing that it is moderately associated with both (Curran et al., 2020; Tang et al., 2011). One recent study used factor analysis to suggest that death anxiety may represent a general factor from which FCR emerges (Berlin & von Blanckenburg, 2022). However, this study used a measure of FOP, rather than FCR, accepting the conflated definition, to demonstrate this factor structure. There are several reasons why death anxiety in FCR and FOP is so under-studied, but the most salient reason is that most theoretical models do not include death anxiety as a central feature in the development or maintenance of FCR or FOP (Sharpe et al., 2018).

Since the first model of FCR was published (Lee-Jones et al., 1997), most contemporary models of FCR and FOP have proposed different, but complimentary, cognitive accounts of the development and maintenance of FCR and FOP. These cognitive models have focused on several different factors, for example risk perception and threat appraisal (Lee-Jones et al., 1997), or metacognitions and intrusive thoughts (Fardell et al., 2016). These cognitive factors have been integrated into the cognitive-processing model of FCR (Fardell et al., 2016). This model proposes that when a cancer patient has unhelpful metacognitions, which are beliefs that worry is uncontrollable, harmful, or helpful. Normal worries about cancer recurrence or progression trigger a chain reaction of worry, rumination, and threat monitoring that drives FCR. However, only one model has posited that existential concerns, like death anxiety, are central to the development of FCR or FOP (Simonelli et al., 2017). This model retains some components of earlier cognitive formulations, but uniquely applied terror management theory to the experience of cancer. According to terror management theory (Pyszczynski et al., 1999), due to the natural inclination to preserve one’s life, awareness of or proximity to death can elicit unbearable anxiety. Hence, when someone is reminded of their mortality, such as when facing the possibility of cancer recurrence, the proximal defences, i.e., avoidance, are invoked to ameliorate death anxiety (Simonelli et al., 2017). This is an understandable reaction that is often effective in reducing anxiety in the short term. However, without adopting a cultural worldview that offers meaning and transcendence beyond death, the so-called distal defences, avoidant coping will eventually reinforce the anxiety and make death more threatening. In the model developed by Simonelli et al. (2017) external reminders of cancer, or physical symptoms that may indicate a recurrence or progression, are filtered through the proximal and distal terror management defences. Those cues not filtered out by these defences are cognitively appraised before eliciting some degree of FCR or FOP (Simonelli et al., 2017).

While Curran et al. found that death anxiety was one of four key predictors of FOP in multiple regression, the individual contribution of death anxiety to the prediction of FOP was not reported (Curran et al., 2020). It is also not known if death anxiety is more related to FCR or FOP. Hence, the present study aims to determine if death anxiety can predict both FCR and FOP in people with ovarian cancer, over and above known theoretical predictors. If FCR is a form of cognitive avoidance, as proposed by Coutts-Bain et al. (2022) we would expect that death anxiety will be more strongly associated with FOP than FCR. Nevertheless, it is expected that death anxiety will account for unique variance in FCR and FOP over and above known predictors.

## Method

### Participants

Two-hundred and seven adults with a previous or current diagnosis of ovarian cancer accessed an online survey in response to advertisements distributed by Ovarian Cancer Australia (OCA). This sample was recruited as part of a larger study investigating FCR in breast and ovarian cancer (Coutts-Bain et al., 2022). Participants were excluded if they did not consent to the survey (*n* = 3), or did not progress beyond the demographic items (*n* = 28). Hence, the final analysed sample consisted of 176 participants living with and beyond ovarian cancer.

### Procedure

Participants were recruited between the 11th of June 2020 and 11th of September 2020 via the OCA email database and social media. These advertisements included a description of the study and a Qualtrics survey link. Eligible participants completed the 20 to 30-minute survey in their own time.

### Measures

#### Demographics

Participants responded to demographic and medical history questions related to age, marital status, children, education, employment, disease and treatment history.

#### Fear of cancer recurrence & fear of progression

The nine item Fear of Cancer Recurrence Inventory (FCR-I) severity subscale was used to measure FCR (Simard & Savard, 2009). Scores range from 0 to 36. Higher scores indicate greater FCR. Internal consistency was α = 0.865.

The 12-item short-form Fear of Progression Questionnaire (FoP-Q-SF; Mehnert et al., 2006) was used to assess FOP. This short-form version has been validated in people with cancer (Mehnert et al., 2006). Scores could range from 12 to 60. Higher scores indicate greater FOP. Internal consistency was α = 0.880.

#### Death anxiety

Death anxiety was assessed using the 15-item Death Anxiety Questionnaire (Conte et al., 1982). This questionnaire assesses a person’s worries about the process of dying and the consequences of death. Scores could range from 0 to 30. Higher scores indicate a greater degree of death anxiety. Internal consistency was α = 0.850.

#### Threat appraisal

Threat appraisals of cancer were assessed using the six item threat subscale of the Appraisal of Life Events Scale (Ferguson et al., 1999). Scale instructions were slightly modified to orient participants towards their cancer experience, as has been done in previous studies, e.g., (Curran et al., 2020). Scores could range from 0 to 30. Higher scores indicating that cancer is appraised as more threatening. Internal consistency was α = 0.882.

#### Intrusive thoughts

Intrusive thoughts about cancer were assessed using the eight item Impact of Event Scale-revised (IES-R) intrusions subscale (Weiss, 2007). This subscale is validated for assessing intrusive thoughts about cancer in people with cancer (Mystakidou et al., 2007). Scores could range from 0 to 32. Higher scores indicate a greater intensity of intrusive thoughts. Internal consistency was α = 0.927.

#### Metacognitions

Metacognitions were assessed with an 18-item subset of the 30-item short-form Metacognitions Questionnaire (MCQ-30; Wells & Cartwright-Hatton 2004). This subset is a composite of the positive belief, negative belief, and need for control subscales, as these subscales are the most strongly related to FCR and FOP (Butow et al., 2015; Cook et al., 2014). Scores could range from 18 to 72. Higher scores indicate a greater severity of maladaptive metacognitions. Internal consistency was α = 0.911.

#### Subjective risk perception

Perceived risk that the participants’ cancer would recur or progress was assessed with a single item adapted from the short-form Concern About Recurrence Questionnaire (Thewes et al., 2015). Participants reported their belief that their cancer would recur or progress on a scale ranging from 0 to 100%.

### Analyses

All data was analysed in SPSS 26. To assess whether the correlation between death anxiety and FOP was significantly stronger than the correlation between death anxiety and FCR, we used a Z-test procedure for comparing dependent correlations (Meng et al., 1992). To assess whether death anxiety could account for unique variance in FCR and FOP we conducted two hierarchical regression analyses: step (1) age, step (2) threat appraisal, intrusive thoughts, metacognitions, perceived risk of recurrence/progression, and step (3) death anxiety.

## Results

Two hundred and seven participants accessed the online survey, with a completion rate of 76.8%. Little’s Missing Completely at Random test suggests that this missing data was not systematically biased and could be justifiably excluded (*χ*^*2*^ = 621.209, *df* = 585, *p* = .145). Demographics were based on a sample of 179 people aged between 22 and 79 (*M* = 56.59, *SD* = 11.83). Sample demographics and cancer treatment details are reported in Table 1.

**Table 1:** Participant Characteristics

	Frequency (*n*)	Percent (%)
Marital status		
Married	103	57.5
Widowed	8	4.5
Divorced	32	17.9
Separated	6	3.4
Never Married	30	16.8
Children^a^	120	67.0
Education		
Incomplete High School	17	9.5
High School	63	35.2
Undergraduate	57	31.8
Postgraduate	42	23.5
Employment^b^	77	43.0
Stage at Diagnosis		
I	41	22.9
II	20	11.2
III	79	44.1
IV	26	14.5
Unknown	23	7.3
Current Cancer Status		
In Treatment	58	32.4
Active Disease	17	9.5
In Remission	104	58.1
Metastatic Cancer	74	41.3
Surgery^c^	173	96.6
Current Pharmacotherapy^d^		
Chemotherapy	38	21.2
Tamoxifen	4	1.9
Olaprarib	14	7.8
Other Hormone Therapy	7	3.9
Other non-Hormone Therapy	26	14.5

*Note.* Percentages calculated by excluding those with missing data. ^a^ those who have at least one child, ^b^ those in current employment, ^c^ those that have received surgical cancer treatment, ^d^ those currently receiving a specific pharmaceutical treatment

### Correlational analyses

FCR was significantly correlated with death anxiety (*r* = .520, *p* ≤ .001), as was FOP (*r* = .651, *p* ≤ .001), this accounts for 27% and 42.4% of the variance in death anxiety, when these variables are considered in isolation, respectively. FCR was also significantly correlated with FOP (*r* = .630, *p* ≤ .001). When accounting for the intercorrelation between FCR and FOP, death anxiety was most strongly associated with FOP (*Z* = -2.59, *p* = .005, 95%CI [-0.049, − 0.352]). Correlations between all other measures are reported in Table 3.

Given the high correlations observed between the predictor variables, we assessed the risk of multicollinearity. When age, threat appraisal, intrusive thoughts, perceived risk, metacognitions, and death anxiety were used to simultaneously predict FCR or FOP, variance inflation factors ranged between 1.163 and 2.039. Hence, multicollinearity was not a concern within the present study, as these values are below the typical cut-off scores of 5 or 10 used to identify risk of multicollinearity (Belsley et al., 2005).

### Hierarchical regression analyses

Table 2 outlines the results of the hierarchical regression analysis for predicting FCR, and FOP. In the FCR model, in step 1, age was not a significant predictor of FCR. Step 2 accounted for 65%. At this step, FCR was significantly predicted by threat appraisal, intrusive thoughts, and risk perception. The final model accounted for 66% of the variance in FCR. At this step, FCR was significantly predicted by threat appraisal (β = 0.260, *p* ≤ .001), intrusive thoughts (β = 0.278, *p* ≤ .001), risk perception (β = 0.480, *p* ≤ .001), and death anxiety (β = 0.137, *p* = .034). However, death anxiety accounted for only 1% of unique variance in FCR, over and above the preceding factors.

In the FOP model, step 1 accounted for 7.6% of variance in FOP, with age being a significant predictor of FOP. Step 2 accounted for 58.6%. At this step, FOP was significantly predicted by age, threat appraisals, intrusive thoughts, metacognitions, and risk perception. The final model accounted for 63.3% of the variance in FOP. At this step, FOP was significantly predicted by age (β = − 0.108, *p* = .040), threat appraisal (β = 0.191, *p* = .002), intrusive thoughts (β = 0.236, *p* ≤ .001), metacognitions (β = 0.124, *p* = .045), risk perception (β = 0.186, *p* ≤ .001), and death anxiety (β = 0.299, *p* ≤ .001), with death anxiety accounting for a significant 4.7% of unique variance in FOP, over and above the preceding factors.

**Table 2:** Correlations between variables entered into the hierarchical regression models

	FCR	FOP	Death Anxiety	Age	Intrusions	Threat Appraisal	Metacognitions	Risk Perception
FCR	–	.630***	.520***	− 0.112	0.630***	0.487***	0.329***	0.648***
FOP		–	0.651***	− 0.230**	.662***	0.550***	0.509***	0.401***
Death Anxiety			–	− 0.274***	.577***	0.488***	0.533***	0.300***
Age				–	− 0.238**	− 0.202**	− 0.093	0.085
Intrusions					–	0.501***	0.510***	0.377***
Threat Appraisal						–	0.400***	0.116
Metacognitions							–	0.179*
Risk Perception								–

Note.*p ≤ .05,**p ≤ .01,***p ≤ .001

**Table 3:** *Hierarchical regression to predict FCR and FOP*

	FCR												
Predictors	Step 1				Step 2				Step 3				
	*B*(SE)	β	R^2^	∆R^2^	*B*(SE)	β	R^2^	∆R^2^	*B*(SE)	β	R^2^	∆R^2^	
Age	− 0.060 (0.047)	− 0.100	0.010	0.010	− 0.006 (0.030)	− 0.010	0.650	0.640***	0.005 (0.030)	0.008	0.660	0.010*	
Threat Appraisal					0.264 (0.052)***	0.288			0.239 (0.052)***	0.260			
Intrusive Thoughts					0.289 (0.061)***	0.311			0.258 (0.062)***	0.278			
Metacognitions					− 0.020 (0.038)	− 0.030			− 0.047 (0.040)	− 0.070			
Perceived Risk					0.106 (0.011)***	0.500			0.102 (0.011)***	0.480			
Death Anxiety									0.158 (0.074)*	0.137			
	FOP												
	*B*(SE)	β	R^2^	∆R^2^	*B*(SE)	β	R^2^	∆R^2^	*B*(SE)	β	R^2^	∆R^2^	
Age	− 0.232 (0.064)***	− 0.276	0.076	0.076***	− 0.126 (0.046)**	− 0.150	0.586	0.510***	− 0.091 (0.044)*	− 0.108	0.633	0.047***	
Threat Appraisal					0.326 (0.079)***	0.252			0.247 (0.077)**	0.191			
Intrusive Thoughts					0.403 (0.093)***	0.308			0.308 (0.091)**	0.236			
Metacognitions					0.198 (0.058)**	0.209			0.117 (0.058)*	0.124			
Perceived Risk					0.068 (0.017)***	0.229			0.055 (0.016)**	0.186			
Death Anxiety									0.484 (0.108)***	0.299			

*p ≤ .05;**p ≤ .01;***p ≤ .001

## Discussion

The present study aimed to determine if death anxiety was a stronger predictor of FOP than FCR, and whether death anxiety could account for unique variance in FCR and FOP over and above other cognitive factors. In support of our hypotheses, correlational analysis demonstrated that death anxiety was moderately associated with both FCR and FOP, but was more strongly related to FOP. Moreover, the hierarchical regression analyses demonstrated that death anxiety accounts for a small, but statistically significant, proportion of variance in FCR and FOP over and above age, metacognitions, threat appraisal, intrusive thoughts, and risk perception. However, it should be noted that death anxiety contributed to only 1% to the variance of FCR once other variables were already accounted for. Nevertheless, the results provide evidence supporting the notion that for people living with and beyond ovarian cancer, death anxiety is a relevant for both FCR, and FOP, although particularly for FOP.

While death anxiety contributed only a small amount to the variance for FCR and FOP (particularly FCR), it should be borne in mind that the hierarchical regression provides a very stringent test of the potential role of death anxiety. That is, theoretical models and empirical research clearly demonstrate that threat appraisal, risk perception, metacognitions and intrusive thoughts are all strong predictors of FCR (Butow et al., 2015; Cook et al., 2014; Coutts-Bain et al., 2022; Fardell et al., 2016), and FOP (Coutts-Bain et al., 2022; Curran et al., 2020). Together, these factors contributed more than 58.6% of the variance in FCR and FOP. As a result, there was relatively little variance left for death anxiety to predict in FCR or FOP. Hence, the estimates of unique variance for which death anxiety contributed is likely to underestimate the importance of death anxiety in FCR and FOP. This is reflected in our univariate analyses, where in isolation, death anxiety accounts for 27% and 42% of the variance in FCR and FOP, respectively.

### Theoretical implications

The finding that death anxiety is a significant predictor of FCR and FOP provides support to the Simonelli et al. (2017) model of FCR and FOP and the Curran et al. (2017) model of cancer-related anxiety. Although the unique contribution of death anxiety to FCR and FOP was, at best, modest, the bivariate correlations showed that death anxiety has large, positive relationships to both fears. Whilst the present cross-sectional study cannot infer causality, it does suggest that FCR and FOP are inextricably linked to fears of death.

The present results are also consistent with the argument that FCR may be a form of cognitive avoidance for thinking about the consequences of recurrence, i.e. progression and death. That worry in generalized anxiety disorder (GAD) is a form of cognitive avoidance has long been posited (Sibrava & Borkovec, 2006). The present analysis has provided further evidence for the role of FCR as a form of cognitive avoidance of FOP and death anxiety. Indeed, the cognitive avoidance conceptualisation may explain several findings of the present study and broader literature. For example, the present study found that death anxiety is associated with FCR and FOP, but that it most strongly predicts FOP. This would be expected if FOP represents a fear of the consequences of recurrence, i.e., death. Secondly, it is well established that worry is a form of cognitive avoidance in GAD and there is evidence that GAD is the most common comorbidity for FCR (Thewes et al., 2013). Thirdly, longitudinal research has demonstrated that FCR actually increases immediately after a cancer recurrence is diagnosed (Savard & Ivers, 2013). Furthermore, qualitative research has identified that many breast cancer survivors endorse cognitive avoidance as a helpful strategy for coping with their cancer-related fears (Thewes et al., 2016). Importantly, this study also found that those with moderate to high levels of FCR tended to report more vivid fears of death and end of life suffering than those with low levels of FCR, while also reporting that they relied on cognitive avoidance to manage these intrusions. Similarly, qualitatively, it was those with clinical FCR in a mixed cancer sample, that tended to report fears of death (Mutsaers et al., 2016). Lastly, recent factor analytic research has proposed that death anxiety is a general factor that accounts for FOP, as measured by the FoP-Q-SF (Berlin & von Blanckenburg, 2022). All of these findings are consistent with the notion that FCR may be a means to cognitively avoid FOP and fears of death. However, in the absence of rigorous longitudinal research that measures FCR, FOP, and death anxiety over time, this account must remain tentative.

### Clinical implications

The current study has demonstrated that death anxiety is a predictor of FCR and FOP. This draws attention to the need to address death anxiety in psychological interventions for FCR or FOP. One recent meta-analysis of cognitive-behavioural therapy (CBT) trials, many of which involved exposure therapy, illustrated that CBT can effectively reduce death anxiety in clinical and non-clinical samples (Menzies et al., 2018). Despite this, most CBT-based interventions for FCR or FOP do not explicitly target or assess death anxiety, as they tend to focus on other cognitive factors (Tauber et al., 2019). There have been a number of interventions for advanced disease that focus on existential issues, such as death anxiety, e.g., (Rodin et al., 2018; Tauber et al., 2019). Death anxiety was most strongly associated with FOP. Hence, psychological interventions that address death anxiety may particularly benefit those who fear progression of cancer. However, as death anxiety still predicted a notable proportion of variance in FCR in univariate analyses, and remained a statistically significant predictor above other cognitive variables, addressing death anxiety may also be relevant to FCR. Moreover, if FCR is indeed a way to cognitively avoid FOP, and thus potentially fears of death, targeting death anxiety may have relevance to both those with active and non-active disease.

Literature on existentialist therapy and imaginal exposure for patients with early-stage disease remains scant. However, one notable exception was a group-based intervention which combined traditional CBT and existentialist therapy techniques to reduce cancer related distress, rather than FCR or FOP specifically (Kissane et al., 2003). However, a recent, and FCR-specific, cognitive-existential group intervention has effectively reduced FCR in a randomized-controlled trial (Lebel et al., 2014). This intervention incorporated numerous CBT strategies, but notably involved repeated imaginal exposure to the participants’ worst-fear, in relation to their FCR. Moreover, one small, exploratory trial has demonstrated that adherence to a similar worst-case scenario homework task was associated with reduced FCR in a small sample of cancer survivors without any additional CBT strategies (Moran et al., 2017). Although these cognitive-existentialist studies did not specify that these imaginal consequences of recurrence were related to death or dying, it does suggest that imaginal exposure may be a tolerable intervention for addressing death anxiety in cancer survivors. Overall, if novel or adapted psychological interventions that target death anxiety enhance reductions in FCR and FOP, then these therapies may be able to better respond to the needs of cancer survivors by improving quality of life, reduce psychiatric morbidity, and reducing related maladaptive health behaviours. Consequently, such interventions yield not only benefit for individuals with a history of cancer, but also broader society but reducing FCR and FOP-related burden on the healthcare system.

### Study limitations

The results of this study must be qualified by several limitations. It is possible that death anxiety may have differing relevance to FCR and FOP in those with a cancer diagnosis that differs in its nature and prognosis from ovarian cancer, such as breast, skin, or lung cancer. However, one study has found death anxiety to be associated with FOP in a mixed, but predominantly breast, cancer sample, (Curran et al., 2020) and another has found death anxiety to be associated with FCR in a mixed cancer sample (Tang et al., 2011). Hence, it is unlikely that the associations between FCR and FOP with death anxiety are ovarian cancer specific.

Secondly, the Death Anxiety Questionnaire used by the present study is a unidimensional assessment of death anxiety (Conte et al., 1982). However, more recently validated assessments of death anxiety are known to distinguish between different factors within death anxiety. As an example, the Death and Dying Distress Scale distinguishes between distress stemming from finitude and the actual process of dying in people with advanced cancer (Shapiro et al., 2021). Whereas the Death Anxiety Beliefs and Behavioural Scale distinguishes between affect, beliefs, and behaviours related to death anxiety (Menzies et al., 2022). Comparing the relationships between FCR, FOP, and these different components of death anxiety may further elucidate how FCR and FOP are similarly or differentially related to death anxiety.

Thirdly, another limitation is that many of the constructs of interest in this study are highly associated. Whilst this could potentially create difficulties in interpretation, such as multicollinearity, our results and previous research (Coutts-Bain et al., 2022) indicates that each construct is sufficiently distinct although, as theoretically predicted, associated with each other.

Lastly, ovarian cancer is a disease that predominantly affects women, so the current study was unable to elucidate the role of gender, if any, on the relationship between death anxiety and FCR or FOP.

## Conclusions

The present study has provided novel data on the significance of death anxiety to the experience of FCR and FOP in people with ovarian cancer. This exploration of the statistical relationships between FCR, FOP, death anxiety, and other cognitive predictors has clearly demonstrated that death anxiety is a key predictor of FCR and FOP, and remains significant even when accounting for age, metacognitions, intrusive thoughts about cancer, threat appraisal, and risk perception. These findings draw attention to the paucity of theoretical and empirical research on death anxiety as it relates to FCR and FOP, relative to other cognitive factors. Moreover, these findings highlight the potential for exposure and existential therapy to enhance existing cognitive-behavioural interventions for FCR and FOP. The integration of these psychological interventions may yield treatments which can further reduce the significant psychological burden of FCR or FOP, particularly in those with uncertain or poor prognoses.

## Data Availability

Individual, but deidentified participant data and a data dictionary will be made available from publication upon request to researchers who aim to use the data in secondary analyses.
